# Impact of Environmental and Disturbance Variables on Avian Community Structure along a Gradient of Urbanization in Jamshedpur, India

**DOI:** 10.1371/journal.pone.0133383

**Published:** 2015-07-28

**Authors:** Sushant Kumar Verma, Thakur Das Murmu

**Affiliations:** 1 Department of Zoology, Guru Ghasidas Vishwavidyalaya, Bilaspur, Chhattisgarh, India; 2 Independent Researcher, Jamshedpur, Jharkhand, India; University of Waikato (National Institute of Water and Atmospheric Research), NEW ZEALAND

## Abstract

Gradient pattern analysis was used to investigate the impact of environmental and disturbance variables on species richness, species diversity, abundance and seasonal variation of birds in and around Jamshedpur, which is one of the fastest growing cities of India. It was observed that avian community structure is highly influenced by the vegetation habitat variables, food availability and human-related disturbance variables. A total of 61 species belonging to 33 families were recorded from the suburban area. 55 species belonging to 32 families were observed in nearby wildland habitat consisting of natural vegetation whereas only 26 species belonging to 18 families were observed in urban area. Results indicated that the suburban habitat had more complex bird community structure in terms of higher species richness, higher species diversity and higher evenness in comparison to urban and wildland habitat. Bird species richness and diversity varied across seasons. Maximum species richness and diversity was observed during spring season in all type of habitat. Most of the birds observed in urban areas were found to belong to either rare or irregular category on the basis of their abundance. The observed pattern of avian community structure is due to combined effect of both environmental and human related disturbance variables.

## Introduction

Various degrees of human settlement result in differentiation of a landscape in to urban, suburban and rural areas. Seventy percent of the world’s population is expected to live in urban areas by the year 2050 [1]. Around 92% of urban population growth will be observed in developing countries during the next twenty years [2]. As the world’s urban population is growing at nearly 1% per annum on average [3] and cities are expanding in naturally species-rich regions [4] particularly in key biodiversity hotspots [5], a better understanding of the pattern of species composition along urbanization gradient is necessary for sustainable urban planning and conservation.

Urbanization is an important factor reducing local vegetation and increasing habitat fragmentation and ultimately leads to environmental problems, including loss of biodiversity [6]. It not only reduces natural vegetation but also degrades the structure and composition of remaining vegetation [7–8]. Birds are suitable to carry out studies on the impact of urbanization on biodiversity as they are highly sensitive to anthropogenic disturbances [9–10]. Urbanization ultimately leads to biological homogenization in terms of avian diversity which posses a threat to the biotic uniqueness of the local ecosystems [11]. Avian species richness and abundance is positively related to native vegetation and vegetation cover, however, negatively related to construction, car rate and pedestrian rate [12].

Studies on the effect of intensifying urbanization i.e. the rural-to-urban gradient on bird diversity has been investigated in many parts of the world and it has been observed that most diverse bird communities are present in habitats with intermediate level of human disturbance. Low avian species richness and high total density or biomass were observed in urban areas in comparison to adjacent natural areas [13–16]. It has been observed that breeding bird diversity is found to decrease with increased urbanization level [17–18] and to be lowest in urban centers [19–20]. Jokimäki & Suhonen [21] and Blair [19] reported greatest avian species richness at intermediate levels of human disturbance. Suburban areas provide suitable conditions for both urban avoiding native species and urban adapted exotics therefore these areas are often seen with maximum species richness of birds compared to adjacent rural and urban settlements [19]. In general, it was observed that habitat conversion has a negative effect on rare and specialist species, while it favours generalists and behaviourally flexible species [22]. Lack of appropriate adaptations to utilize available resources is the main reason of species loss from urban environments [23]. Such comparative studies on species richness and abundance of birds with various degree of urbanization is lacking in developing nations and therefore needs further investigations in rapidly growing countries like India to study ecological effects of urbanization on avian community.

India’s bird diversity contains 13% of the world species richness, approximately 1300 species [24]. In addition to it Indian Peninsula contains several Endemic Bird Areas. Urbanization is taking place at a faster rate in India and according to a survey by UN State of the World Population report in 2007, by 2030, 40.76% of country's population is expected to reside in urban areas [25]. In spite of this, studies on the effect of urbanization on avian community are not common in this part of the world and mostly confined towards preliminary surveys on avian species richness and abundance of existing natural ecosystems [26–28]. Birds respond well to the availability of habitat structures [17] and ecological effect of urbanization can be well studied by using them as models [29]. Therefore the rural to urban gradient was employed to investigate the differences in avian community pattern due to environmental and disturbance variables along a settlement gradient in and around the city Jamshedpur (India). As environmental factors are greatly influenced by weather conditions it is hypothesized that seasonality of bird occurrence may depend on environmental variables which strongly influence species diversity and richness patterns in different habitats along urbanization gradients. Such type of study may be useful in identification of most vulnerable species of the studied area [8] as well as useful to conservation planners, urban planners, and land managers.

## Materials and Methods

### Study area

Jamshedpur is the largest city in the state of Jharkhand, India. It has been predicted as the 84^th^ fastest growing city in the world for the timeframe 2006–2020 with a population of 1.1 million [30]. It is a planned industrial city which includes more than 1,200 small and medium scale industries. The average elevation of the city is 135 meters. Total geographical area of Jamshedpur is 149.23 km square. The city falls under deciduous type of forest region and the green cover is estimated to be around 33% of the total land area.

Jamshedpur has tropical wet and dry climate. Summer start in April and extend up to June. The temperature variation during summer is from 35 to 44°C while the temperature during winters may drop to 8°C. The city receives rain by south-west monsoon mainly during the month from July to October. The average annual rainfall is about 1200 mm.

The avian diversity of city Jamshedpur (India) with reference to urbanization gradient was studied for two years. Definitions of urban (percent of built-up area > 50, building density > 10/ha, and residential human density > 10/ha), Suburban (percent of built-up area 30–50, building density < 2.5/ha, and residential human density 1–10/ha) and Wildland (percent of built-up area 0–2, building density 0, and residential human density < 1/ha) habitats follow the suggestion made by Marzluff et al. as shown in Table 1 [18]. Percent of built-up area and building density were calculated from Google Earth image whereas residential human density was determined by general demographic survey. The calculated data is presented in Table 2. Approximately 2 km^2^ area was surveyed in each habitat type for calculation of percent of built-up area, building density, and residential human density.

**Table 1:** Terms that describes major land types along urbanization gradient [18].

	Percent built	Building density	Residential human density
**Wild land**	0–2	0	<1/ha
**Suburban**	30–50	<2.5/ha	1–10/ha
**Urban**	>50	>10/ha	>10/ha

**Table 2:** Percent built, building density and residential human density of chosen urban, suburban and wild land habitat in Jamshedpur, India.

	Percent built	Building density	Residential human density
**Wild land**	0	0	0
**Suburban**	40	1.7/ha	7/ha
**Urban**	91	87/ha	212/ha

Wildlands are unsettled lands that may sometimes include dwellings. Suburban lands are characterized by moderate- to high-density, single-family housing often containing lawns and gardens. On the other hand urban lands are areas with high building density.

### Bird surveys

Point count method was used for bird survey. Point counts were conducted in three habitat types along transects using distance sampling [31]. Transects did not cross habitat types and were randomly situated in each habitat type. At each habitat three 1-km transects were used. Five permanent sampling points at 200 m intervals along the long axis of each defined transect was established in all three types of habitats. Transects were sufficiently separated (about 500 m) to avoid double counting of birds. Points were placed along transect in area with natural vegetation or wildland with 2–3m openings in the canopy and in suburban and urban areas along roads with similar sized canopy openings to avoid biased counts [32]. During the study transects were visited once a month. Birds were recorded visually and aurally within 50 m around each sampling point for 5 min to maximize count efficiency. The time of sampling was between 7:00–11:30 am. Each observed bird was recorded and was identified with the help of field guides [33–34].

Only resident bird species of the studied area [28] that regularly used the resources of the respective area for foraging or nesting during the study period were included. Nocturnal birds were excluded from the observations. Relative abundance was calculated by a ratio of the number of individuals observed for each species to the total number of individuals observed.

On the basis of their monthly mean abundance data obtained, bird species were categorized under following four categories following Ramírez-Albores & Ramírez [35] as these categories are most useful when used flexibly rather than rigidly:

1. Abundant (total of 40 or more individuals recorded),
2. Common (17 to 39 individuals recorded),
3. Scarce (11 to 16 individuals recorded),
4. Irregular (five to 10 individuals recorded) and
5. Rare (one to four individuals recorded).

Taxonomy adopted here is after Inskipp et al. [36].

### Environmental variables

Environmental variables of interest in this study were mainly related to vegetation variables which include percentage cover of vegetation, number of vegetation layer, percent of bare soil and percent of leaf litter and woody debris as previously selected by Heileman et al. [37]. Insects and fruit plants constitute important food sources for birds therefore they were counted as a measure of their food irrespective of species. Every type of vegetation present within the range of 50 m radius around each point count was analyzed for the determination of relative abundance of insects and to determine the availability of fruit plants for birds. To determine relative abundance of insects, 05 trees, 10 shrubs and 20 herbs were randomly selected. 5 branches of each tree and shrub were randomly selected whereas whole herb was scrutinized. An ordinal rank abundance score (0 = no insect, 1 = 1–15 insects, 2 = 16–30 insects, 3 = 30+ insects) was assigned to each point count and average was calculated for each level of urbanization. Similarly 20 trees and 20 herbs were randomly selected for determination of availability of fruit plants. An ordinal rank abundance score (0 = absence of fruit plants, 1 = 1–5 fruit plants, 2 = 6–15 fruit plants, 3 = 16+ fruit plants) was assigned to each point count and average was calculated for each level of urbanization. Obtained monthly values of species richness and abundance for all three types of habitats were polled and then compared by season (Spring: February to march; Summer: April to June; Rainy season: July to October; Winter: November to January).

### Disturbance variables

The degree of human-related disturbance at each count point site was measured by quantifying vehicle and pedestrian traffic and cover of built structure according to Heileman et al. [37]. Vehicle and pedestrian traffic levels were scaled as 1 = low (less than five vehicles and/or pedestrians per minute), 2 = moderate (5–10 vehicles and/or pedestrians per minute) or 3 = high (greater than 10 vehicles and/or pedestrians per minute) at each count point by visual estimation at the time of the bird surveys. Percentage cover of human built structure was also determined around 50 m of each count point.

### Data analysis

No parameters such as species richness and species diversity showed significant differences between years so we pooled the data across years for data analysis. Bird species diversity and bird species richness were measured using Shannon-Wiener diversity index (H = -Σ p_i_.ln.p_i_) and Margalef’sindex [(S-1)/ln(n)], respectively [38]. We chose Margalef’s species richness index for its ease of calculation and its widespread use [38].

Avian community structure comparison between the different habitat types was estimated by Morisita index of similarity, as this index is nearly independent of sample size and it is mended as one of the best overall measures of similarity for ecological use [39].

For comparing species richness, species diversity and relative abundance of individuals between habitats, a two way ANOVA was used. To compare relative abundance only those species were taken in to consideration which were common between habitat types. Two way ANOVA was also used to test the seasonal variation in species richness and diversity across study areas.

The species richness between habitats were compared by using individual based rarefaction curves [40] which describe species richness while controlling for the confounding effect of sampling effort and bird density [41]. To estimate species richness non-parametric estimators of Chao 1, Chao 2, Jacknife I and Bootstrap was used. Hurlbert’s [42] probability of interspecific encounter (PIE) was also calculated for each habitat class. PIE controls for both sampling effort and bird density, and uses repeated re-sampling of the data to calculate the probability that the next bird sampled will be of a different species. Therefore, high PIE values indicate high species evenness. Estimate S (Version 9.1.0) was used to obtain the rarefaction curves and the species richness estimators, after randomizing the sample 100 times [43]. Hurlbert’s [42] probability of interspecific encounter (PIE) was obtained through Ecosim.

Overall species richness and abundance of birds in all three types of habitats were compared with environmental and human related disturbance variables using two way ANOVA tests.

### Ethics statement

Observation of birds does not require ethics approval or legal permits because it does not involve any important effect on animal welfare. Surveys were conducted in areas which are open to the public, therefore there was no need to ask land managers for approval.

## Results

**Fig 1:**
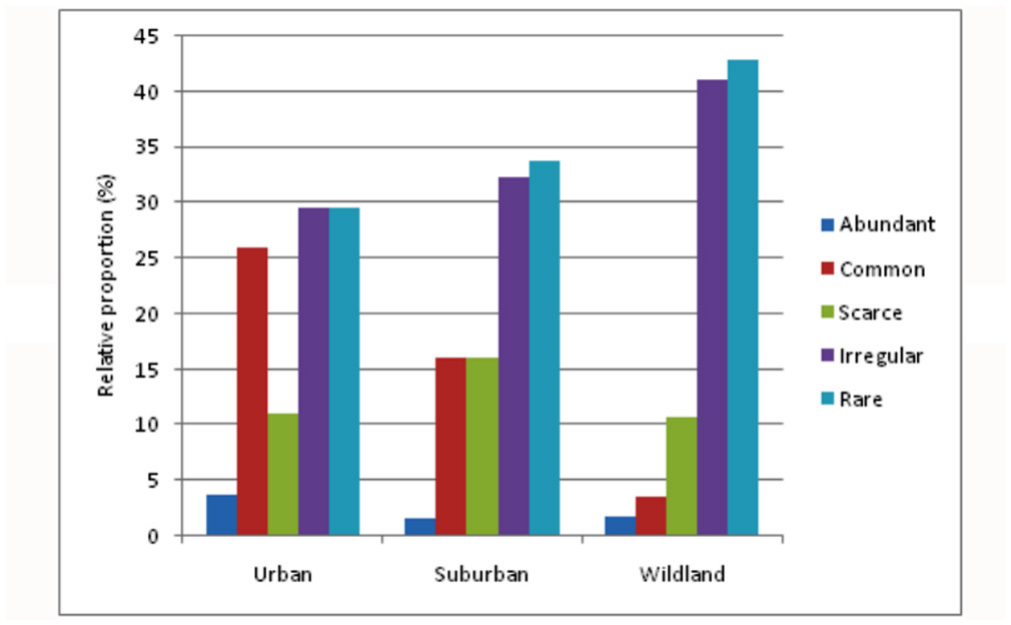
Graph showing the relative proportion (%) of different avian species in all studied habitat on the basis of abundance category.

Overall 65 species of birds belonging to 34 families were recorded during the study period. The maximum species richness was observed in suburban area (61 species from 33 families) followed by wild land area (55 species from 32 families) with urban being taxonomically poorest (26 species from 18 families). A checklist has been prepared showing the observed species in each habitat along with its abundance category (Table 3). The relative proportion (%) of different avian species in all studied habitat on the basis of abundance category is shown in Fig 1.

**Table 3:** Check List of Birds observed at different selected sites (UR-Urban, SU-Suburban, WL- Wild land).

S.N	Family	Common Name	Scientific Name	UR	SU	WL
				A	A	A
1	*Accipitridae*	Black-shouldered Kite	*Elanus caeruleus*	0	I	I
2	*Alaudidae*	Ashy-crowned Finch-lark	*Eremopterix grisea*	0	R	R
3	*Alcedinidae*	Common Kingfisher	*Alcedo atthis*	0	I	0
4		White-throated Kingfisher	*Halcyon smyrnensis*	0	R	0
5	*Anatidae*	Gadwall	*Anas strepera*	0	C	C
6		Lesser Whistling-duck	*Dendrocygna javanica*	C	C	C
7		Comb Duck	*Sarkidiornis melanotos*	S	S	S
8	*Ardeidae*	Indian Pond-heron	*Ardeola grayii*	R	S	R
9		Eastern Cattle Egret	*Bubulcus coromandus*	I	I	R
10		Intermediate Egret	*Mesophoyx intermedia*	0	R	0
11		Black-crowned Night-heron	*Nycticorax nycticorax*	0	R	R
12	*Burhinidae*	Indian Stone-curlew	*Burhinus oedicnemus*	0	0	R
13	*Capitonidae*	Coppersmith Barbet	*Megalaima haemacephala*	I	I	I
14	*Charadridae*	Grey-headed Lapwing	*Vanellus cinereus*	0	R	R
15		Red-wattled Lapwing	*Vanellus indicus*	0	S	0
16	*Cisticolidae*	Ashy Prinia	*Prinia socialis*	I	I	I
17	*Columbidae*	Rock Pigeon	*Columba livia*	A	C	I
18		Spotted Dove	*Streptopelia chinensis*	I	I	I
19		Eurasian Collared-dove	*Streptopelia decaocto*	0	R	I
20		Laughing Dove	*Streptopelia senegalensis*	0	R	R
21	*Coraciidae*	Indian Roller	*Coracias benghalensis*	0	I	R
22	*Corvidae*	Indian Jungle Crow	*Corvu smacrorhynchos*	0	R	I
23		House Crow	*Corvus splendens*	C	C	0
24		Rufous Treepie	*Dendrocitta vagabunda*	0	R	I
25	*Cuculidae*	Greater Coucal	*Centropus sinensis*	C	R	R
26		Asian Koel	*Eudynamys scolopacea*	I	I	R
27		Common Hawk-cuckoo	*Hierococcyx varius*	0	R	R
28	*Daniidae*	Brown Shrike	*Lanius cristatus*	0	R	I
29		Black-headed Long-tailed Shrike	*Lanius schach tricolor*	0	R	R
30	*Dicruridae*	Ashy Drongo	*Dicrurus leucophaeus*	0	I	R
31		Black Drongo	*Dicrurus macrocercus*	I	I	R
32	*Estrildidae*	Indian Silverbill	*Lonchura malabarica*	0	R	I
33	*Accipitridae*	Black Kite	*Milvus migrans*	C	I	I
34	*Jacanidae*	Pheasant-tailed Jacana	*Hydrophasianus chirurgus*	0	R	0
35		Bronze-winged Jacana	*Metopidius indicus*	0	R	0
36	*Meropidae*	Little Green Bee-eater	*Merops orientalis*	S	C	S
37	*Motacicillidae*	Paddyfield Pipit	*Anthus rufuls*	0	C	I
38	*Muscicapidae*	Oriental Magpie-Robin	*Copsychus saularis*	S	0	0
39		Red-breasted Flycatcher	*Ficedula parva*	0	0	R
40		Indian Black Robin	*Saxicoloides fulicata*	0	S	R
41		Jungle Babbler	*Turdoides striatus*	0	C	A
42	*Nectariniidae*	Purple Sunbird	*Nectarinia asiatica*	I	I	I
43	*Oriolidae*	Indian Golden Oriole	*Oriolus kundoo*	0	I	I
44	*Phalacrocoracidae*	Little Cormorant	*Phalacrocorax niger*	0	0	R
45	*Phasianidae*	Grey Francolin	*Francolinus pondicerianus*	0	I	I
46		Indian Peafowl	*Pavo cristatus*	0	I	S
47	*Picidae*	Woodpecker	*Dendrocopos*	R	R	R
48	*Ploceidae*	Scaly-breasted Munia	*Lonchura punctulata*	R	S	I
49		House Sparrow	*Passer domesticus*	C	C	0
50		Indian Baya Weaver	*Ploceus philippinus*	R	C	0
51	*Podicipitidae*	Little Grebe	*Tachybaptus ruficollis*	0	I	R
52	*Psittacidae*	Alexandrine Parakeet	*Psittacula eupatria*	0	S	S
53		Rose-ringed Parakeet	*Psittacula krameri*	R	S	R
54	*Pycnonotidae*	Red-vented Bulbul	*Pycnonotus cafer*	I	S	I
55		Red-whiskered Bulbul	*Pycnonotus jocosus*	R	R	I
56	*Rallidae*	White-breasted Waterhen	*Amaurornis phoenicurus*	R	S	I
57		Common Moorhen	*Gallinula chloropus*	0	R	R
58	*Scolopacidae*	Common Sandpiper	*Actitis hypoleucos*	0	R	R
59		Green Sandpiper	*Tringa ochropus*	0	I	R
60	*Sturnidae*	Bank Myna	*Acridotheres ginginianus*	0	S	I
61		Common Myna	*Acridotheres tristis*	C	A	I
62		Asian Pied Starling	*Sturnus contra*	C	C	S
63		Grey-headed Starling	*Sturnus malabaricus*	0	R	R
64		Brahminy Starling	*Sturnus pagodarum*	0	I	S
65	*Upupidae*	Common Hoopoe	*Upupa epops*	0	I	I

‘A’ represents their abundance category (R = rare, I = irregular, S = scarce, C = common and A = abundant, 0 = absent).

**Fig 2:**
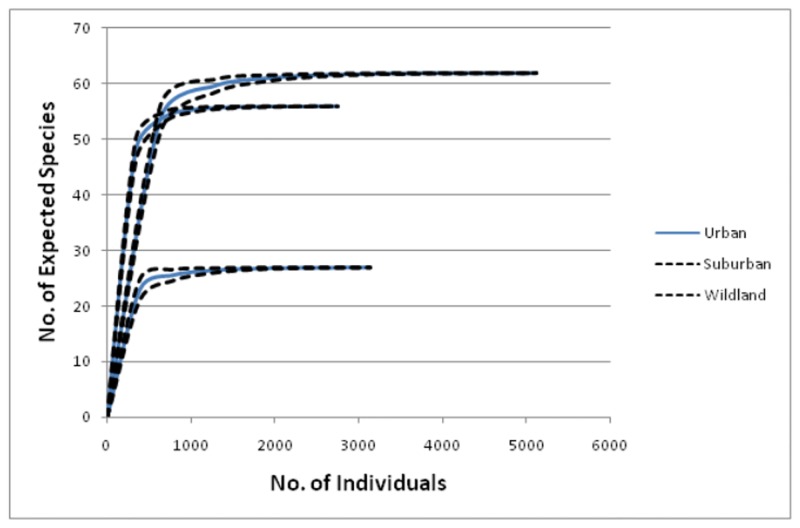
Rarefaction curves for avian species estimated from urban, suburban and wild land habitat for city Jamshedpur. Thick lines indicate mean richness and dotted lines indicate 95% confidence intervals.

**Fig 3:**
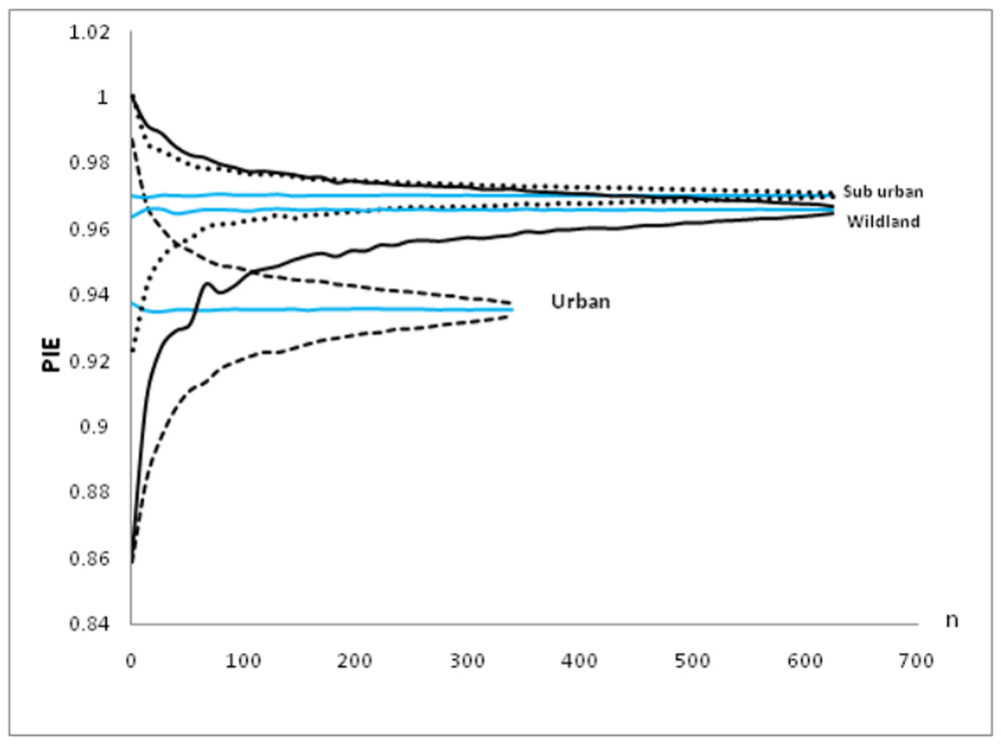
Species evenness shown by probability of interspecific encounter (PIE) for each habitat class (PIE is shown by thick lines whereas dotted lines show 95% confidence intervals).

Margalef’s index and Shannon-Wiener diversity index for different habitats representing various levels of urbanization is shown in Table 4. Analysis revealed that suburban habitat had significantly higher species richness and diversity than urban habitat: high Margalef’s index (F = 520.57, P< 0.0001), high Shannon-Wiener diversity index (F = 91.11, P< 0.0001). However no significant difference in species richness was observed between suburban and wildland habitat: Margalef’s index (F = 0.2 P = 0.659), but species diversity showed a significant difference: Shannon-Wiener diversity index (F = 5.33, P = 0.030). Species rarefaction curves (Fig 2) from different habitats confirmed that suburban habitat had a higher number of avian species than wild land and urban habitats. High PIE value was obtained for suburban area (Fig 3) which is an indicative of high evenness followed by wild land and urban area.

**Table 4:** Number of observed species (S_ob_), Margalef’s index (±SE) and Shannon-Wiener diversity index (± SE) for different habitats representing various levels of urbanization obtained through line transect and point count method of field survey.

	S_ob_	Margalef’s index	Shannon-Wiener diversity index
**Urban**	26	3.802 ± 0.13 ^a^	2.708 ± 0.090^1^
**Sub-urban**	55	8.533 ± 0.16 ^b^	3.608 ± 0.259^2^
**Wild-land**	61	8.420 ± 0.28 ^b^	3.485 ± 0.046^3^

Significant difference in Margalef’s index between habitats is shown with different alphabetic (lowercase) superscripts while that of Shannon-Wiener diversity index with different numeric superscripts.

Morisita index of similarity showed greater similarity between bird communities at suburban and wildland habitat than urban and wildland or urban and suburban habitat (Table 5).

**Table 5:** Comparison of avian community of urban, suburban and wildland habitat by Morisita index of similarity.

	Suburban	Wild land
**Urban**	0.286	0.299
**Sub-urban**		0.598

Non-parametric estimators of chao I, chao II, first order Jackknife mean and bootstrap mean of urban, suburban and wildland bird species are shown in Table 6.

**Table 6:** Non-parametric estimators of chao I, chao II, first order Jackknife mean and bootstrap mean of urban, suburban and wildland bird species.

	Urban	Suburban	Wildland
**Chao I mean**	30.41± 1.21	62.90± 1.43	58.41±2.46
**Chao II mean**	32.64±0.91	71.70±2.42	66.88± 1.10
**Jacknife I mean**	41.82± 1.10	77.16±3.11	69.97± 1.85
**Bootstrap mean**	39.47±0.98	72.66± 1.71	70.18±0.91

Relative abundance of common and top 22 species which are present in greater proportion in all types of studied habitat was calculated and compared (Table 7). Out of total 65 species, 22 species shared urban, suburban and wildland habitat and 16 out of them differs significantly across habitat types. Relative abundance data clearly indicated that abundance of some species like *Columba livia*, *Centropus sinensis*, *Acridotheres tristis*, *Sturnus contra and Milvus migrans* increases with urbanization.

**Table 7:** Relative abundance of common and top 22bird species recorded across three habitats.

Species	Relative Abundance			Significant Difference
	Urban	Suburban	Wildland	
*Dendrocygna javanica*	5.49	4.26	7.85	F = 5.17, P = 0.011138
*Sarkidiornis melanotos*	2.96	1.69	2.93	
*Ardeola grayii*	0.26	1.75	0.46	F = 258.34, P < .0001
*Bubulcus coromandus*	1.32	0.92	0.51	F = 34.12, P < .0001
*Megalaima haemacephala*	1.48	1.01	1.60	
*Prinia socialis*	1.60	0.97	1.63	
*Columba livia*	14.68	5.37	1.67	F = 716, P < .0001
*Streptopelia chinensis*	1.48	0.99	1.60	
*Centropus sinensis*	8.08	0.24	0.39	F = 578.34, P < .0001
*Eudynamys scolopacea*	1.36	1.04	0.49	F = 49.05, P < .0001
*Dicrurus macrocercus*	1.30	1.00	0.35	F = 58.67, P < .0001
*Milvus migrans*	7.33	0.90	1.70	F = 416.22, P < .0001
*Merops orientalis*	2.85	4.73	3.04	F = 159.36, P < .0001
*Nectarinia asiatica*	1.52	0.97	1.67	
*Dendrocopos*	0.32	0.21	0.60	
*Lonchura punctulata*	0.45	1.76	1.77	F = 118.23, P < .0001
*Psittacula krameri*	0.30	1.94	0.60	F = 283.27, P < .0001
*Pycnonotus cafer*	1.50	1.84	1.81	F = 65.31, P < .0001
*Pycnonotus jocosus*	0.47	0.26	1.79	F = 29.5, P < .0001
*Amaurornis phoenicurus*	0.32	1.87	1.79	F = 133.69, P < .0001
*Acridotheres tristis*	8.36	4.23	1.74	F = 1683.38, P < .0001
*Sturnus contra*	8.40	5.16	3.04	F = 462.47, P < .0001

Bird species richness and diversity fluctuated across seasons for all habitat types as shown in Table 8 and Table 9 respectively. Maximum species richness was observed during spring while minimum was obtained during summer. Species richness differs significantly between urban and suburban (P< 0.0001), urban and wildland (P< 0.0001) and suburban and wildland (P = 0.0117) in spring. Species richness also differs in summer, rainy and winter between urban and suburban (P< 0.0001) and urban and wildland (P< 0.0001). However no such difference was observed between suburban and wildland habitat during summer, rainy and winter season. Species richness also significantly varies between seasons for urban (P = 0.0010), suburban (P = 0.0010) and wildland (P = 0.0075). On the other hand bird species diversity differs significantly between urban and suburban (P< 0.0001), urban and wildland (P< 0.0001) in spring. In summer it differs significantly between urban and suburban (P = 0.0215), urban and wildland (P = 0.042) and between suburban and wildland (P = 0.002). In rainy season species diversity differs between urban and suburban (P< 0.0001), urban and wildland (P< 0.0001) and between suburban and wildland (P = 0.020). During winter species diversity significantly differs between urban and suburban (P< 0.0001), urban and wildland (P< 0.0001) and suburban and wildland (P = 0.002). Species diversity also significantly varies between seasons for suburban (P< 0.0001) and wildland (P< 0.0001). However no such difference in species diversity was observed for urban habitat (P = 0.151).

**Table 8:** Seasonal variation in avian species richness measured through Margalef’s index in different studied habitats.

	Urban	Suburban	Wildland
**Spring**	4.209 ± 0.10^a1^	9.156 ± 0.01^b1^	8.972 ± 0.04^c1^
**Summer**	3.133 ± 0.20^a2^	7.796 ± 0.26^b2^	7.515 ± 0.43^b2^
**Rainy**	3.998 ± 0.05^a3^	8.474 ± 0.04^b3^	8.469 ± 0.05^b3^
**Winter**	3.967 ± 0.09^a3^	8.705 ± 0.09^b4^	8.725 ± 0.03^b4^

Values with different alphabetic (lowercase) superscripts differ significantly between habitats within a season. Values with different numeric superscripts differ significantly between seasons within a particular habitat.

**Table 9:** Seasonal variation in avian species diversity measured through Shannon-Wiener diversity index in different studied habitats.

	Urban	Suburban	Wildland
**Spring**	2.899 ± 0.01^a1^	3.738 ± 0.01^b1^	3.694 ± 0.04^b1^
**Summer**	2.362 ± 0.31^a2^	3.501 ± 0.01^b2^	3.278 ± 0.03^c2^
**Rainy**	2.786 ± 0.01^a3^	3.606 ± 0.01^b3^	3.514 ± 0.02^c3^
**Winter**	2.785 ± 0.03^a3^	3.587 ± 0.03^b3^	3.454 ± 0.01^c3^

Values with different alphabetic (lowercase) superscripts differ significantly (*p* < 0.01) between habitats within a season. Values with different numeric superscripts differ significantly (*p* < 0.01) between seasons within a particular habitat.

Avian species diversity is highly influenced by the environmental and human related disturbance variables in all types of studied habitats (Table 10). It was found to be positively related with total percent vegetation cover, number of vegetation layer, percent of bare soil and percent of leaf litter and woody debris. However it was negatively related with vehicle and pedestrian traffic and cover of built structure. Significant difference (*p* < 0.01) in ordinal rank score for insects and fruit yielding plants was obtained between sub urban and urban as well as between sub urban and wild land habitat but the difference was insignificant (*p* > 0.01) between urban and wild land habitat.

**Table 10:** Relationship between avian species richness and various environmental and human related disturbance variables in urban, suburban and wildland habitats.

Sl. No	Variables	Species richness
1	Vegetation % cover	0.778***
2	No. of Layers	0.695***
3	Soil	0.546**
4	Leaf litter/woody debris	0.675***
5	Built Environment	- 0.991***
6	Vehicle Traffic	- 0.886***
7	Pedestrian Traffic	- 0.891***
8	Insects	0.987***
9.	Fruit yielding plants	0.942***

** = P<0.01.

*** = P<0.001.

## Discussion

The two-year study on bird diversity in relation to urbanization gradient in Jamshedpur, India revealed that the urbanization has a clear impact on the avian community. It was observed that the distribution of most of the species varied across the urban gradient with peak at one level and decreased with either greater or lesser intensity of urbanization.

Results confirmed that suburban habitat had higher avian species richness, species diversity and abundance than wildland and urban habitat. The obtained result is similar to those obtained by Jokimäki & Suhonen [21] and Blair [19] who also reported greater avian species richness and diversity at intermediate level of human disturbance.

The obtained pattern of avian community structure along urbanization is due to joint effect of environmental and human related disturbance variables. Present study indicated that several vegetation habitat variables significantly contribute towards avian community structure. Increase in vegetation cover increases the species diversity [14]. High vegetation cover was obtained for both suburban and wild land areas where avian species richness is more in comparison to urban areas. This high vegetation cover attract both human-commensal as well as high proportion of native bird species [44] resulting in high avian diversity [45]. In addition to vegetation cover, other habitat variables like: number of vegetation layers, percentage of soil and percentage of leaf litter on land also plays important role in deciding the avian species richness of a habitat. More number of vegetation layer, high percentage of soil and leaf litter on land in suburban area in comparison to wildland and urban habitat creates a mosaic habitat which can attract non-native species to penetrate from the wild land and nearby urban areas and ultimately resulted in increased species richness and diversity as compared to urban and wild land habitats [46–47].

Availability of food is an important determining factor for the presence or absence of a species from a particularly locality. Insects and fruits are important food component of birds [48]. In the present study high degree of association was observed between species richness and abundance of insects and fruit plants in all types of studied habitat. Although large amount of food is available in urban settings in the form of human refuse but are of low quality which may reduce the survival rate of nestlings [49]. Due to high vegetation cover and greater availability of fruit yielding plants, suburban landscapes provide continuous food supply to birds and thus support high diversity of birds.

Results indicated that avian species richness is negatively related with human disturbance variables like vehicle and pedestrian traffic and cover of built structure as previously reported by other workers [50–51]. Noise due to vehicle traffic affects reproductive behavior of many birds and ultimately their survival [52]. Vocalization is an important tool in birds to attract mates as well as to defend their territory. The increased noise level due to heavy traffic in urban habitats can interfere with their vocal communication [53]. Due to increased noise level house finches started to produce low frequency songs with decreased number of notes per song [54] which resulted in decreased mating opportunities as females prefer males producing longer songs [55]. In rapidly developing cities like Jamshedpur one cannot ignore the role of noise pollution produced from vehicle traffic for low avian species richness. The obtained vehicle and pedestrian traffic levels clearly supported the above observation in our study also. Built environment reduces vegetation cover and thus reproductive ability of all those species which prefers to build nest on herbs and trees. No such parameters related to nesting was studied in the present study however less vegetation cover obtained for the urban habitat supports the above hypothesis.

Suburban and wild land area showed greater similarity value as represented in terms of Morisita index. Many species are common for both habitats. Suburban areas are provided with patches of natural vegetation which supports species of wild land habitat also.

Seasonal species richness varies in all type of habitat which are mainly due impact of environmental factors which might cause the seasonal movements of birds within and between habitats [56]. In spring maximum species richness occurs while summer shows minimum species richness. Seasonal variation in diversity index value differs significantly between habitats in each season as well as during different seasons in each level of urbanization. Many bird species depends on insects as their food source. Thus their availability in a particular type of habitat depends indirectly on invertebrate availability. Low temperatures generally limit invertebrate activity and reproduction, while heavy rainfall may inhibit invertebrate activity and the foraging efficiency of birds. Thus seasonality of bird occurrence strongly influenced the avian community along urbanization gradient.

The proportion of rare and irregular species dominated in all types of studied habitat. Very few species belongs to abundant and common category. It indicates that the bird community within the city is comparatively less than its adjoining area so initiatives must be taken to conserve them. We strongly suggest and recommend that the demographic studies on birds and their relation to landscape complexity should be carried out in the developing cities for their conservation. As a result of development processes nearby wildland habitat as well as garden, parks and other barren lands inside the city are increasingly converted in to residential or market areas and ultimately creating adverse condition for native birds to survive in such degraded habitats.

From conservation point of view it becomes necessary to create special feeding niche in urban settings so that species can coexist. Plantation of fruit plants can additionally provide food for many avian species can become one of the most important factor to attract nearby wildland dwelling birds or previously urban dwellers to become inhabitant of urban areas again. Establishment of considerable feeding stations at various parts of the cities can encourage dominant species to increase the foraging chances for subordinate species which ultimately help them to increase their population and contribute towards maintenance of evenness.

High avian species richness in suburban areas may be due to occurrence of natural vegetation in small patches which help in conserving species with isolated distribution [57]. Therefore it is highly recommended to incorporate small parks and garden areas in urban management systems. Artificial aquatic systems should be integrated into urban areas for conservation of avian diversity.

## References

[1] United Nations World Urbanization Prospects: The 2007 Revision Population Database, 2007. Available: http://esa.un.org/unup/.

[2] Carr D. Rural migration: The driving force behind tropical deforestation on the settlement frontier. Prog Hum Georg. 2009; 1:33(3): 355–378. 10.1177/0309132508096031 PMC2872490 20485541

[3] United Nations World population prospects: the 2010 revision. 2010 New York, NY: UN.

[4] Luck GW. A review of the relationships between human population density and biodiversity. Biol Rev. 2007; 82: 607–45. 17944620 10.1111/j.1469-185X.2007.00028.x

[5] Seto KC, Guneralp B, Hutyra LR. Global forecasts of urban expansion to 2030 and direct impacts on biodiversity and carbon pools. Proc Nat Acad Sci, USA. 2012; 109: 16083–16088. 22988086 10.1073/pnas.1211658109 PMC3479537

[6] Grimm NB, Faeth SH, Golubiewski NE, Redman CL, Wu J, Bai Z, et al Global change and the ecology of cities. Science. 2008;319: 756–760. 10.1126/science.1150195 18258902

[7] Germain SS, Rosenstock SS, Schweinsburg RE, Richarsdson WS. Relationships among breeding birds, habitat, and residential development in Greater Tucson, Arizona. Ecol Appl. 1998; 8:680–691.

[8] Marzluff JM, Gehlbach FR, Manuwal DA. Urban environments: influences on avifauna and challenges for the avian conservationist In: Marzluff JM, Sallabanks R, editors.Avian conservation: research and management; 1998 Island Press, Washington, DC, USA.

[9] Chazdon RL, Peres CA, Dent D, Sheil D, Lugo AE, Lamb D, et al The potential for species conservation in tropical secondary forests. Conserv Biol. 2009; 23: 1406–1417. 10.1111/j.1523-1739.2009.01338.x 20078641

[10] Gibson L, Lee TM, Koh LP, Brook BW, Gardner TA, Barlow J, et al Primary forests are irreplaceable for sustaining tropical biodiversity. Nature. 2011; 478: 378–383. 10.1038/nature10425 21918513

[11] McKinney ML. Urbanization as a major cause of biotic homogenization. Biol Conserv. 2009; 127 (3):247–260.

[12] Villegas M, Zavala AG. Bird community responses to different urban conditions in La Paz, Bolivia. Urb Eco. 2010; 13: 375–391.

[13] Crooks KR, Suarez AV, Bolger DT. Avian assemblages along a gradient of urbanization in a highly fragmented landscape. Biol Conser. 2004; 115: 451–462.

[14] Sandstrom UG, Angelstam P, Mikusinski G. Ecological diversity of birds in relation to the structure of urban green space. Land Urb Plan. 2005; 77:39–53.

[15] Chapman KA, Reich PB. Land use and habitat gradients determine bird community diversity and abundance in suburban, rural and reserve landscapes of Minnesota, USA. Biol Conserv. 2007; 135: 527–541.

[16] Caula S, Marty P, Martin JL. Seasonal variation in species composition of an urban bird community in Mediterranean France. Land Urb Plan. 2008; 87: 1–9.

[17] Clergeau P, Savard JPL, Mennechez G, Falardeau G. Bird abundance and diversity along an urban-rural gradient: a comparative study between two cities on different continents. Condor. 1998; 100(3):413–425.

[18] Marzluff JM. Worldwide urbanization and its effects on birds In: Marzluff JM, Bowman R, Donnelly R, editors.Avian ecology in an urbanizing world;2001 Kluwer Academic, Norwell, Massachusetts, USA.

[19] Blair RB. Land use and avian species diversity along an urban gradient. Ecol Appl. 1996; 6(2):506–519.

[20] Tratalos J, Fuller RA, Evans KL, Davies RG, Newson SE, Greenwood JJD, et al Bird densities are associated with household densities. Global Change Biol. 2007; 13(8):1685–1695.

[21] Jokimaki J, Suhonen J. Effects of urbanization on the breeding bird species richness in Finland: A biogeographical comparison. Ornis Fennica. 1993; 70: 71–77.

[22] Moller AP. Successful city dwellers: a comparative study of the ecological characteristics of urban birds in the Western Palearctic. Oecologia. 2009; 159:849–858. 10.1007/s00442-008-1259-8 19139922

[23] Sol D, González-Lagos C, Moreira D, Maspons J, Lapiedra O. Urbanisation tolerance and the loss of avian diversity. Ecol Lett. 2014; 17: 942–950. 10.1111/ele.12297 24835452

[24] Grimmett R, Inskipp C, Inskipp T. Birds of the Indian subcontinent. Oxford University Press, New Delhi, India;1998.

[25] United Nations. State of world population 2007: Unleashing the Potential of Urban.2007. Available: http://www.unfpa.org.

[26] Mattu VK, Thakur ML. Bird Diversity and Status in Summer hill, Shimla (Himachal Pradesh). Indian Forest. 2006; 132 (10):1271–1281.

[27] Thakur ML, Mattu VK, Lal H, Sharma V, Raj H, Thakur V. Avifauna of Arki Hills, Solan (Himachal Pradesh), India. Indian Birds. 2010; 5 (6): 162–166.

[28] Verma SK. A preliminary survey on the avian community of Dalma Wildlife Sanctuary, Jharkhand, India. J threat taxa. 2011; 3(5): 1764–1770.

[29] McDonnell MJ, Hahs AK. The use of gradient analysis studies in advancing our understanding of the ecology of urbanizing landscapes: current status and future directions. Landsc Ecol. 2008; 23: 1143–1155.

[30] City Mayors “World’s fastest growing urban areas (1)” 2014. Available: http://www.citymayors.com/statistics/urban_growth1.html.

[31] Styring AR, Ragai R, Unggang J, Stuebing R, Hosner PA, Sheldon FH. Bird community assembly in Bornean industrial tree plantations: Effects of forest age and structure. Forest Ecol Manag. 2011; 261:531–544.

[32] Hutto RL, Hejl SJ, Kelly JF, Pletschet SM. A comparison of bird detection rates derived from on-road versus off-road point counts in northern Montana In: Ralph CJ, Sauer JR, Droege S, editors. Monitoring bird population by point counts.U.S. Dept. Agric. For. Serv. Gen. Tech. Rep. PSW-GTR-149; 1995 pp. 103–110.

[33] Kazmierczak K, Singh R. A Bird Watcher’s Guide to India. Oxford University Press 2001.

[34] Ali S. The Book of Indian Birds—13th Edition Bombay Natural History Society/Oxford University Press; 2001.

[35] Ramírez-Albores JE, Ramírez G. Avifauna de la egionoriente de la sierra de Huautla, Morelos, México. Anales Inst Biol Univ Nac Autón Méx Ser Zool. 2002; 73: 91–111.

[36] Inskipp T, Lindsey N, Duckworth W. An Annotated Checklist of the Birds of the Oriental Region. Oriental Bird Club, Sandy, UK; 1996.

[37] Heileman MR, McQuillan PB, Kirkpatrick JB. Habitat effects on bird groups along an urban gradient in Hobart, Tasmania. 2007. Available: https://wikis.utas.edu.au/download/attachments/12852121/Bushland%20Birds_draft.pdf.

[38] Magurran AE. Measuring biological diversity. Oxford: Blackwell Science; 2004.

[39] Krebs CJ. Ecological Methodology.2nd ed–Menlo Park, California, Addison-Welsey Publishers;1999.

[40] Colwell RK, Mao CX, Chang J. Interpolating, extrapolating and comparing incidence-based species accumulation curves. Ecology. 2004; 85: 2717–2727.

[41] Gotelli NJ, Entsminger GL. Ecosim: Null models for ecology version 6.0 (computer program) Acquired Intelligence Inc. and Kesey-Bear. Available: http://homepages.together.net/,gentsmin/ecosim.html.2001.

[42] Hurlbert SH. The non concept of species diversity: a critique and alternative parameters. Ecology. 1971; 52: 577–585. 28973811 10.2307/1934145

[43] Colwell RK. Estimates: Statistical estimation of species richness and shared species from samples. Version 9.User's Guide and application available: http://purl.oclc.org/estimates. 2013.

[44] Daniels GD, Kirkpatrick JB. Does variation in garden characteristics influence the conservation of birds in suburbia? Biol Conserv. 2006; 133: 326–335.

[45] Blair RB. Birds and butterflies along an urban gradient: Surrogate taxa for assessing biodiversity? Ecol Appl. 1999; 9:164–170.

[46] Soderstrom B, Part T. Influence of landscape scale on farmland birds breeding in semi-natural pastures. Conserv Biol. 2000; 14:522–533.

[47] Burke DM, Nol E. Landscape and fragment size effects on reproductive success of forest-breeding birds in Ontario. Ecol Appl. 2000;10: 1749–1761.

[48] Bhullar S, Majer J. Arthropods on street trees: a food resource for wildlife. Pacific Conserv Biol. 2000; 6: 171–173.

[49] Sauter A, Bowman R, Schoech SJ, Pasinelli G. Does optimal foraging theory explain why suburban Florida scrub-jays (*Apheloco macoerulescens*) feed their young human-provided food? Behav Ecol Sociobiol. 2006; 60:465–474.

[50] Fernandez-Juricic E. Avifaunal use of wooded streets in an urban landscape. Conserv Biol. 2000; 106: 129–136.

[51] Reijnen R, Foppen R, terBraak C, Thissen J. The effects of car traffic on breeding bird populations in woodland. III. Reduction of density in relation to the proximity of main roads. J Appl Ecol. 1995;32: 187–202.

[52] Halfwerk W, Holleman L J M, Lessells C M, Slabbekoorn H. Negative impact of traffic noise on avian reproductive success. J App Ecol. 2011; 48: 210–219

[53] Warren PS, Katti M, Ermann M, Brazel A. Urban bioacoustics: It’s not justnoise. Ani Behav. 2006;71:491–502.

[54] Fernández-Juricic E, Poston R, De Collibus K, Morgan T, Bastain B, Martin C, et al. Microhabitat selection and singing behavior patterns of male house finches (*Carpodacus mexicanus*) in urban parks in a heavily urbanized landscape in the western U.S. Urban Habitats, 2005. Available: http://www.urbanhabitats.org/v03n01/finch_full.html.

[55] Nolan PM, Hill GE. Female choice for song characteristics in the house finch. Ani Behav. 2004; 67:403–410.

[56] Norris DR, Marra PP. Seasonal interactions, habitat quality and population dynamics in migratory birds. Condor. 2007;109: 535–547.

[57] Schwartz MW. Choosing the appropriate scale of reserves for conservation. Ann Rev Ecol Syst. 1999; 30: 83–108.

